# Rhomboid intercostal block versus serratus block for postoperative analgesia after thoracoscopic sympathectomy for primary palmar hyperhidrosis: a randomized controlled trial

**DOI:** 10.1186/s12871-023-02203-z

**Published:** 2023-07-19

**Authors:** Mohamed G. Elhouty, Khaled Elbahrawy, Mostafa S. Elawady

**Affiliations:** Faculty of Medicine, Mansoura University, Mansoura, Egypt

**Keywords:** Rhomboid intercostal block, Serratus anterior plane block, Thoracoscopy

## Abstract

**Background:**

Although thoracoscopic sympathectomy is made via small incisions, it is associated with severe postoperative pain. Both Rhomboid intercostal block (RIB) and serratus anterior plane block (SABP) are recent techniques used for pain control after such procedures. Herein, we compared RIB and SAPB regarding pain control in patients undergoing thoracoscopic sympathectomy for palmar hyperhidrosis.

**Patients and methods:**

Three groups were enrolled in this prospective randomized study (71 patients in each group); Group S received SAPB, Group R received RIB and Group C as controls. The block procedures were performed after general anesthesia and prior to the skin incision.

**Results:**

The three groups showed comparable demographics and operative time (*P* ˃ 0.05). Pain scores showed a significant decline with the two block procedures compared to controls during the first day following surgery (both *P* ˂ 0.05), but Group R had better scores compared to Group S. Both block techniques were associated with a significant prolongation of the time to first rescue analgesic and less fentanyl consumption compared to controls (both *P* ˂ 0.05). However, both parameters were improved with RIB rather than SAPB (both *P* ˂ 0.05). Both blocks led to a significant improvement in patient satisfaction than in the control group (both P ˂ 0.05), but it was comparable between the two approaches (*P* ˃ 0.05).

**Conclusion:**

Both RIB and SAPB are safe and effective in pain reduction after thoracoscopic sympathectomy procedures in patients with hyperhidrosis. Moreover, RIB is superior to SAPB as it is associated with better analgesic outcomes.

**Trial registration:**

Pan African Trial Registry PACTR202203766891354. https://pactr.samrc.ac.za/Researcher/TrialRegister.aspx?TrialID=21522

## Introduction

Primary palmar hyperhidrosis is a disorder characterized by chronic excessive sweating despite the absence of sweating triggers. With the evolution of surgical approaches, upper thoracic dorsal sympathectomy performed via video-assisted thoracoscopic surgery (VATS) is the main management option for that clinical entity [1].

Although VATS is performed through small incisions, postoperative pain remains challenging, as some patients report sharp chest pain with deep inspiration, while others report constant pain in the dorsal region. This might necessitate prescribing opioid analgesia for proper pain relief [2].

Postoperative pain not only increases patients' anxiety but also decreases postoperative satisfaction and hinders early rehabilitation, so effectively preventive post-operative pain is important [3]. Different techniques have been described, including paravertebral block, thoracic epidural analgesia, intercostal nerve block, and local wound infiltration [4, 5].

Disadvantages of the previously mentioned techniques are infection, hematoma parasympathetic manifestations, paraspinal muscular pain, spinal anesthesia, and short duration of analgesia [6, 7].

Recently, rhomboid intercostal block (RIB) and serratus anterior plane block (SAPB) have been described to provide effective analgesia after thoracic procedures [8, 9]. RIB was described in 2016 as an alternative to thoracic epidural analgesia [10]. The local anesthetic agent is delivered into the plane between the rhomboid major and intercostal muscles. That provides good analgesia for the anterior and posterior hemithorax [11]. SAPB induces blocking of the lateral cutaneous intercostal nerves, and its efficacy has been proved after thoracotomy and mastectomy procedures. Local anesthesia is instilled, either superficial or deep, in relation to the serratus muscle [12, 13]. Therefore, we conducted this study to compare RIB and SAPB regarding pain control in patients undergoing thoracoscopic sympathectomy for palmar hyperhidrosis.

### Patients and methods

This prospective randomized controlled parallel study was conducted over one year, starting from March 2022 to March 2023, at Mansoura university hospitals after the Institutional Research Board and ethical committee of our university (MFM-IRB) approval (R.21.09.1462) on 31/10/ 2021. The study was designed for 213 patients with severe palmar hyperhidrosis after explaining its benefits and potential risks. Patients were interviewed, and written informed consent was obtained. All methods were performed in accordance with the declaration of Helsinki. The study was registered in the Pan African Trial Registry (PACTR202203766891354) on 16/3/2022. The study was reported according to the Consolidated Standards for Reporting Trials (CONSORT) guidelines[14]. 

The sample size was calculated via the PASS software program (version 23.0.2.), considering the time to the first rescue analgesic as the main outcome. The null hypothesis was considered as the absence of difference between RIB and SAPB techniques regarding that parameter. No previous studies have compared these two specific block techniques before. A minimal patient sample of 64 patients was required in each group to achieve a power and significance level of 80% and 5%, respectively. With an expected seven patients to drop out, the included sample was increased to 71 in each group (a total number of 213 cases).

Patients aged between 15 to 40 years old, from either gender, scheduled for VATS, and classified as class I or II according to the American Society of Anesthesiologists (ASA) were included. Contrarily, we excluded patients whose ASA was more than II, patients with infection at the injection site, bleeding disorders, and psychiatric illnesses.

### Randomization and blinding

A total of 213 patients were randomly assigned to three equal groups using a computer-generated table of random numbers: Group R included patients who received RIB, Group S included patients who received SAPB, and Group C included the remaining patients who received no block procedure. The group allocation was concealed in sequentially numbered, sealed, and opaque envelopes. This study was double-blinded, as patients, data collectors, and those assessing the outcomes (a trained nurse who was not involved in the study), were blinded to the allocation until the end of the trial.

Before the procedure, proper history-taking, clinical examination, and laboratory investigations were performed. All patients were also taught how to express their pain via the Visual analog scale (VAS), which ranges from 0 to 10, with the former for no pain and the latter for the worst pain ever[15].

### Patient's data

At the operative theater, all patients were sedated by IV midazolam 2 mg, and standard monitoring was applied. General anesthesia was induced by IV propofol, atracurium, and fentanyl with doses of 2.5 mg/kg, 0.5 mg/kg, and 1 μg/kg, respectively. A single-lumen endotracheal tube was used. All patients were kept on pressure-controlled volume mode, with a 1:2 I/E ratio and 4 ml/kg tidal volume. The respiratory rate was adjusted to keep ETCO2 around 35 mmHg. Maintenance of anesthesia was done via a mixture of isoflurane and 50% air in oxygen, and atracurium (0.1 mg/kg) increments were administered when needed. The block procedures were performed before skin incision under aseptic conditions under ultrasound guidance. We used Philips Clear Vue 350, WA, USA) with a high-frequency linear transducer (6 – 12 MHz).

**Fig. 1:**
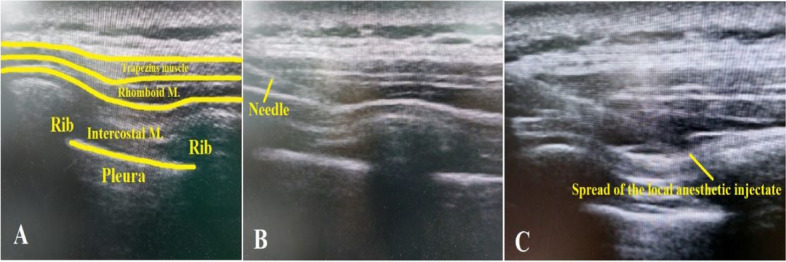
The rhomboid block technique: **(A)** Sonographic anatomy of the layers encountered during rhomboid block. **(B)** Directing the needle to the plane between intercostal and rhomboid muscles. **(C)** Spread of the local anesthetic injectate in the proper plane

In Group R, RIB was done in the lateral position, with the operating side directed superiorly with the abduction of the ipsilateral arm. The linear ultrasound probe was positioned in the oblique sagittal plane medial to the medial scapular border, and the following structures were identified; rhomboid muscle, trapezius muscle, intercostal muscles, pleura, and lungs. Then, a 21-gauge sono-visible needle was inserted at the level of T 5–6 till reaching the plane between the rhomboid and intercostal muscles, where 20 ml of bupivacaine 0.25% was injected. The spread of the local anesthetic was noted by ultrasound, and the procedure was repeated for the opposite side after adjusting the patient's position (Fig. 1).

**Fig. 2:**
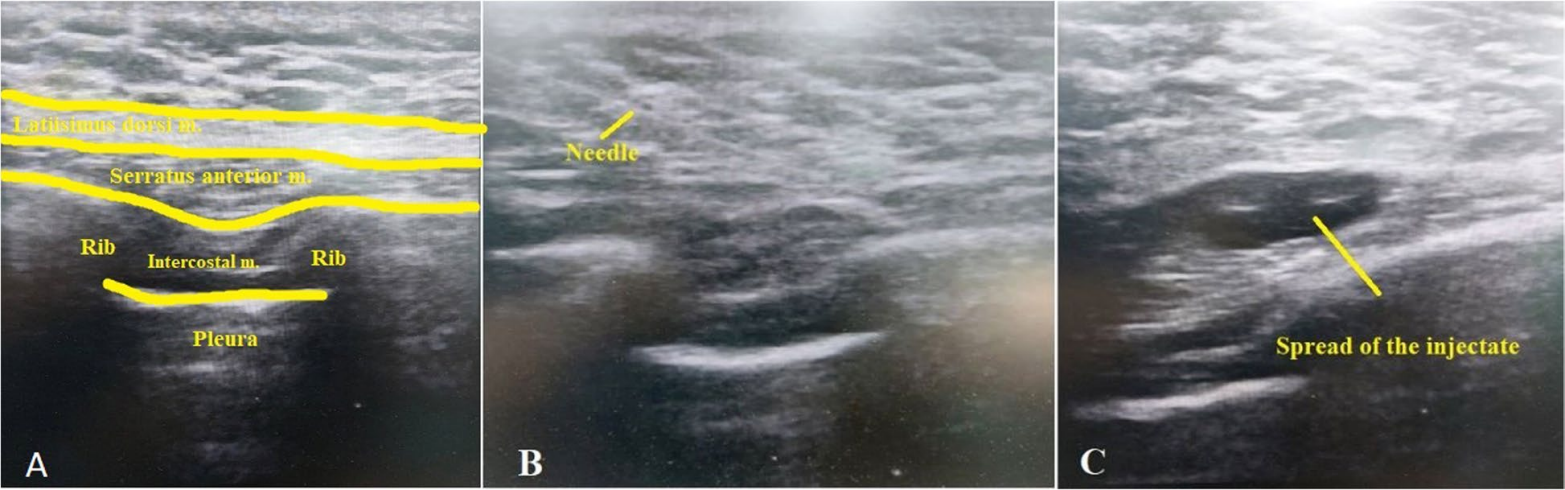
The serratus anterior block technique: **(A)** Sonographic anatomy of the layers encountered during serratus block. **(B)** Directing the needle to the plane between serratus and latissimus dorsi muscles. **(C)** Spread of the local anesthetic injectate in the proper plane

In Group S, SABP was performed in the lateral position. The probe was positioned in the sagittal plane over the thoracic region in the midaxillary line, where the seventh rib was identified along with the following muscles; serratus anterior, latissimus dorsi, and teres major. The sonovisible 18-gauge needle was advanced till reaching the plane superficial to the serratus muscle, where 20 ml of bupivacaine 0.25% was injected. The procedure was repeated for the opposite side (Fig. 2).

No block procedure was done in Group C, as patients in this group received conventional IV analgesia. Heart rate (HR) and mean arterial pressure (MAP) were recorded basally, with skin incision, and then every 15 min till the procedure ended. At the end of the procedure all patients received acetaminophen with doses of 1 mg/kg.

Patients were transferred to the recovery room where VAS score was recorded immediately, 1, 3, 6, 12, and 24 h after the operation. Postoperative analgesia was achieved by IV ketorolac (30 mg/12 h) and IV acetaminophen (1 gm/8 h). IV fentanyl (1 μg/kg) was also commenced for breakthrough pain, and it was repeated after 4 h till the patient had a VAS of 3 or less. Time to the first rescue analgesia and total postoperative opioid consumption was also recorded. Patient satisfaction was graded by the same person(trained nurse) according to a five-point Likert scale; weak, medium, good, very good, and excellent [16].

### Collected data

The primary outcome was the time to the first rescue analgesic, whereas secondary ones included intraoperative hemodynamic changes, pain scores, postoperative opioid consumption, the incidence of complications, and patient satisfaction.

### Statistical analysis

Statistical analysis was performed by the SPSS (version 21.0; SPSS Inc, Chicago, Ill, United States) software program. Numerical data were expressed as mean and standard deviation and compared between the three groups using the one-way ANOVA test. The categorical ones were expressed as numbers and percentages and compared between the three groups using the Chi-square test or Fisher's exact test. Post-hoc analysis (Tukey pairwise comparison) was also performed to compare each individual two groups. P-values less than 0.05 were considered significant. P was used to express the comparison between the three groups. P1, P2, and P3 were used to compare Groups R and S, Groups R and C, and Groups S and C, respectively, as shown in the following tables in the results section.

## Results

**Fig. 3:**
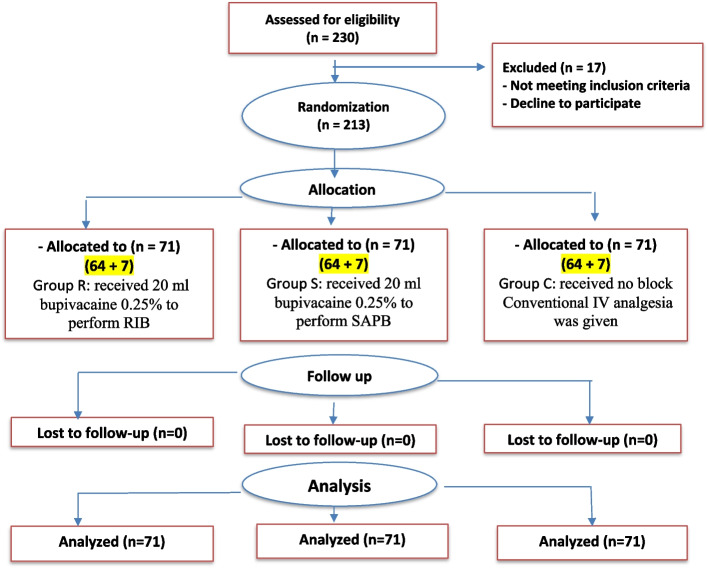
CONSORT Flow Chart

The current study assessed 230 patients for eligibility; 17 patients were excluded. Subsequently, 213 patients assigned for thoracoscopic sympathectomy were randomized to receive RIB (Group R), SAPB (Group S), and Group C included the remaining patients who received no block procedure. No cases were lost to follow up. Thus, the data of 213 patients were analyzed (Fig. 3).

The age of the included patients had mean values of 23.17, 24.38, and 23.69 years, whereas their weight had mean values of 79.37, 80.66, and 78.34 kg in Groups R, S, and C, respectively. Most cases in the three groups were ASA I classification with percentages of 66%, 63%, and 65% of cases, respectively, whereas the remaining cases had class II. The duration of the procedure had an average of 47.68, 50.14, and 48.89 min in the same three groups, respectively. There was no significant difference regarding age, body weight, physical status, and duration of the surgery among the three studied groups (Table 1).

**Table 1:** Demographic characteristics and duration of the thoracoscopic procedure in the three study groups

		Group R (*n* = 71)	Group S (*n* = 71)	Group C (*n* = 71)	P	P1	P2	P3
Age (years)		23.17 ± 4.254	24.38 ± 4.327	23.69 ± 4.194	0.238	0.092	0.467	0.335
Weight (kg)		79.37 ± 14.31	80.66 ± 13.42	78.34 ± 11.33	0.573	0.559	0.64	0.293
ASA	**I**	66 (93.0%)	63 (88.7%)	65 (91.5%)	0.667	˃ 0.05	˃ 0.05	˃ 0.05
	**II**	5 (7.0%)	8 (11.3%)	6 (8.5%)				
Duration of surgery (minutes)		47.68 ± 10.685	50.14 ± 10.986	48.89 ± 9.727	0.376	0.163	0.492	0.477

Data is expressed as mean and standard deviation or as percentage and frequency. P is significant when ˂ 0.05. P1: Group R vs Group S. P2: Group R vs Group C. P3: Group S vs Group C

**Fig. 4:**
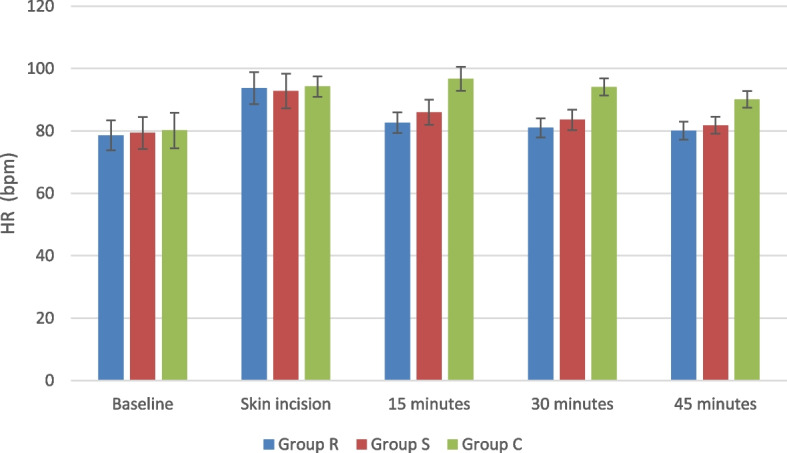
Heart rate changes in the study groups: *R group*Rhomboid intercostal block group, *S group* Serratus anterior plane block group, *C group* Control group

**Fig. 5:**
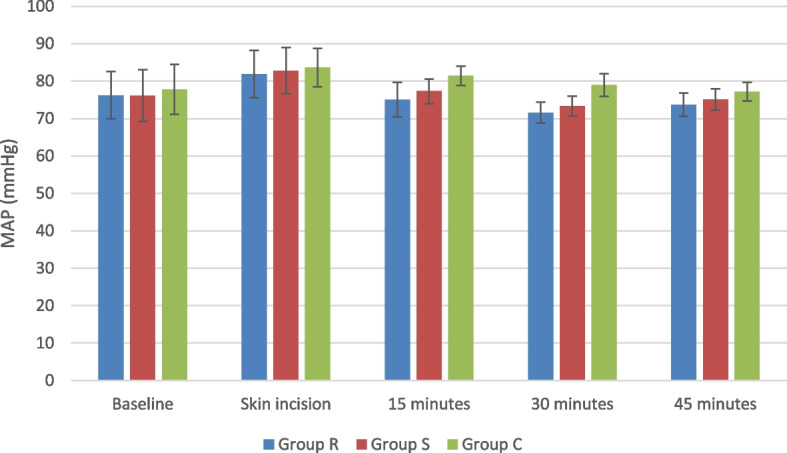
MAP changes in the study groups: *R group*Rhomboid intercostal block group, *S group* Serratus anterior plane block group, *C group* Control group

Both HR and MAP showed no significant difference between the three study groups at the baseline and with the skin incision. Nonetheless, the two block groups expressed significantly lower readings after 15 min till the end of the procedure compared to Group C. This decline in both vital parameters was more evident in Group R compared to Group S (Fig. 4) (Fig. 5).

VAS score was significantly lower in both R and S groups compared to Group C starting from PACU admission till 24 h postoperatively. VAS score was superior in Group R compared to Group S in most of the recorded readings, apart from the PACU and 24-h readings, that were comparable between the two blocks (Table 2).

**Table 2:** Visual analog scale changes in the three study groups

VAS	Group R (*n* = 71)	Group S (*n* = 71)	Group C (*n* = 71)	P	P1	P2	P3
PACU	0.00 ± 0.00	0.11 ± 0.318	4.37 ± 0.541	˂ 0.001**	0.196	˂ 0.001**	˂ 0.001**
One hour	0.00 ± 0.00	0.23 ± 0.453	1.35 ± 0.758	˂ 0.001**	0.027*	˂ 0.001**	˂ 0.001**
Three hours	0.00 ± 0.00	1.30 ± 0.641	2.68 ± 0.752	˂ 0.001**	˂ 0.001**	˂ 0.001**	˂ 0.001**
Six hours	0.65 ± 0.510	2.21 ± 1.027	4.20 ± 1.142	˂ 0.001**	˂ 0.001**	˂ 0.001**	˂ 0.001**
12 h	2.42 ± 0.966	3.83 ± 0.793	3.27 ± 0.894	˂ 0.001**	˂ 0.001**	˂ 0.001**	˂ 0.001**
24 h	3.73 ± 0.716	3.93 ± 0.834	4.24 ± 0.620	˂ 0.001**	0.108	˂ 0.001**	0.012*

Data is expressed as mean and standard deviation. P was used to compare the three groups. P1: Group R vs Group S. P2: Group R vs Group C. P3: Group S vs Group C

^*^: *p* value < 0.05, **: *p* value < 0.001

The time to the first rescue analgesic showed a significant decline in Group C (40.07 min) compared to the other two groups (730.7 min and 562.39 min in Groups R and S, respectively). Additionally, Group C showed a significant increase in fentanyl consumption (107.99 µg) compared to the other two block groups (47.11 and 66.55 µg in Groups R and S, respectively). Group R showed significantly better outcomes compared to Group S regarding both the first analgesic request and fentanyl consumption. Patient satisfaction showed no significant difference between R and S groups, and it was significantly better than group C (Table 3). No block-related complication was encountered in the three studied groups.

**Table 3:** Time of the first analgesic request, opioid consumption, and patient satisfaction in the three study groups

		**Group R (*n* = 71)**	**Group S (*n* = 71)**	**Group C (*n* = 71)**	**P**	**P1**	**P2**	**P3**
**1**^**st**^** Request of analgesia (minutes)**		730.70 ± 56.65	562.39 ± 60.39	40.07 ± 16.551	˂ 0.001**	˂ 0.001**	˂ 0.001**	˂ 0.001**
**Fentanyl (µg)**		47.11 ± 8.479	66.55 ± 16.916	107.99 ± 18.89	˂ 0.001**	**˂ 0.001**	˂ 0.001**	˂ 0.001**
**Satisfaction**	**Weak**	0 (0.0%)	0 (0.0%)	19 (26.8%)	˂ 0.001**	˃ 0.05	˂ 0.05*	˂ 0.05*
	**Medium**	0 (0.0%)	0 (0.0%)	23 (32.4%)				
	**Good**	15 (21.1%)	28 (39.4%)	26 (36.6%)				
	**Very good**	35 (49.3%)	32 (45.1%)	3 (4.2%)				
	**Excellent**	21 (29.6%)	11 (15.5%)	0 (0.0%)				

Data is expressed as mean and standard deviation or as percentage and frequency. P is significant when ˂ 0.05. P1: Group R vs Group S. P2: Group R vs Group C. P3: Group S vs Group C

^*^: *p* value < 0.05, **: *p* value < 0.001

## Discussion

The current study demonstrates that both RIB and SAPB were effective techniques in providing analgesia for patients undergoing VATS. RIB and SAPB were performed before the skin incision, "preemptive analgesia," to induce better analgesia compared to blocks performed after surgery owing to its protective effect on the human nociceptive system [17].

Both block techniques were effective in pain control after the procedure, and the analgesic profile was much better in RIB (rejecting our null hypothesis). That could be explained by the extent of the block in both techniques. RIB covers the entire hemithorax anteriorly and posteriorly, while SAPB covers the anterolateral chest wall region as it mainly affects the lateral intercostal nerve branches and omits the posterior main sensory branch [11, 18].

Our finding correlate with those of Zhang et al., who conducted a prospective trial comparing RIB, SAPB, and erector spinae plane block (ESPB) in patients undergoing VATS. Postoperative pain scores, the time to first analgesic request, and opioid consumption were comparable between RIB and ESPB and more superior to SAPB [19].

Similar results have been reported in a retrospective study compared RIB to SAPB in VATS procedures and found that pain scores were significantly reduced with RIB. Nonetheless, opioid consumption was statistically comparable between the two block techniques (122 mg vs. 151 mg in Groups R and S, respectively – *p* > 0.05) [20].

RIB was initially described by Elsharkawy and his associates. The local anesthetic agent is administered into the fascial plane between the rhomboid and intercostal muscles, providing analgesia for the region supplied by T 2–9 dermatomes in both anterior and posterior chest walls by targeting the lateral cutaneous branches of the ventral thoracic nerves. The spread of the local anesthetic in both craniocaudal and anteroposterior directions could explain its wide coverage, even when the injectate is administered into a single level [11, 21].

SAPB was first described by Blanco and his associates for pain management after chest and breast operations [18]. Serratus muscle is superficial, easily identified, and considered a landmark for thoracic wall blocks as intercostal nerves pierce it. The block procedure provided sufficient analgesia extending from T2 to T9 dermatomes [22, 23].

As regard RIB, our finding was in agreement with Deng et al., who evaluated RIB for elective unilateral VATS. The total amount of sufentanil in 24 h after the surgery in the RIB group was less than those in the control group at (*p* < 0.001). Time to first analgesic request in group RIB was significantly prolonged in the RIB group than those in the control group (*p* < 0.001) [24].

Similar results have been reported by Altıparmak et al., who demonstrated that RIB was associated with a significant improvement in patient recovery (recovery score 164.8 vs. 153.5 in controls – *p* < 0.001) and Postoperative morphine consumption (5 vs. 10 mg in controls – *p* < 0.001) [25].

As regard SAPB, our findings correlate with those of Park et al., who evaluated SAPB on postoperative pain and opioid consumption after thoracoscopic surgery. SAPB reduced the worst median (IQR [range]) pain score 6 (5–7 [3–10]) vs. 7 (6–7 [3–10]), *p* = 0.027. Also, fentanyl consumption was significantly reduced in the block group for the first 24 h after thoracoscopic surgery (3.8 vs. 5.7 μg/kg in controls) [26].

Moreover, Chen and his associates evaluated the effects of SAPB on postoperative pain after thoracoscopic surgery compared with local anesthetic (LA) infiltration. Postoperative sufentanil consumption in the SAPB group was significantly lower compared with the LA group (*P* < 0.01). The amount of rescue analgesia also decreased significantly in the SAPB group (*P* = 0.02) during 0–12 h [27].

There are limitations of our study as the patients were collected from a single medical center, both block techniques were performed as a single shot, and its effect on chronic postoperative pain was not evaluated. That is why we recommend performing more studies with a larger patient sample.

## Conclusion

Both RIB and SAPB are safe and effective in pain management after VATS procedures for hyperhidrosis. However, RIB is superior to SAPB as it is associated with better analgesic outcomes.

## Data Availability

The individual participant data will be available upon reasonable request with the corresponding author after the local IRB approval.

## References

[1] Wolosker N, Faustino CB, de Campos JRM, Kauffman P, Yazbek G, Fernandes PP, et al. Comparative analysis of the results of videothoracoscopic sympathectomy in the treatment of hyperhidrosis in adolescent patients. J Pediatr Surg. 2020;55(3):418–24.10.1016/j.jpedsurg.2019.11.004. 10.1016/j.jpedsurg.2019.11.004 32063368

[2] Neustein SM, McCormick PJ. Postoperative analgesia after minimally invasive thoracoscopy: what should we do? Can J Anaesth. 2011;58(5):423–5, 5–7.10.1007/s12630-011-9475-9. 10.1007/s12630-011-9475-9 21347738

[3] Dastan F, Langari ZM, Salamzadeh J, Khalili A, Aqajani S, Jahangirifard A. A comparative study of the analgesic effects of intravenous ketorolac, paracetamol, and morphine in patients undergoing video-assisted thoracoscopic surgery: A double-blind, active-controlled, randomized clinical trial. Ann Card Anaesth. 2020;23(2):177–82.10.4103/aca.ACA_239_18. 10.4103/aca.ACA_239_18 PMC7336963 32275032

[4] Bédat B, Abdelnour-Berchtold E, Perneger T, Licker MJ, Stefani A, Krull M, et al. Comparison of postoperative complications between segmentectomy and lobectomy by video-assisted thoracic surgery: a multicenter study. J Cardiothorac Surg. 2019;14(1):189.10.1186/s13019-019-1021-9. 10.1186/s13019-019-1021-9 PMC6836384 31699121

[5] Taketa Y, Irisawa Y, Fujitani T. Comparison of ultrasound-guided erector spinae plane block and thoracic paravertebral block for postoperative analgesia after video-assisted thoracic surgery: a randomized controlled non-inferiority clinical trial. Reg Anesth Pain Med. 2019.10.1136/rapm-2019-100827. 10.1136/rapm-2019-100827 31704789

[6] Yeung JH, Gates S, Naidu BV, Wilson MJ, Gao Smith F. Paravertebral block versus thoracic epidural for patients undergoing thoracotomy. Cochrane Database Syst Rev. 2016;2(2):Cd009121.10.1002/14651858.CD009121.pub2. 10.1002/14651858.CD009121.pub2 PMC7151756 26897642

[7] Sheets NW, Davis JW, Dirks RC, Pang AW, Kwok AM, Wolfe MM, et al. Intercostal Nerve Block with Liposomal Bupivacaine vs Epidural Analgesia for the Treatment of Traumatic Rib Fracture. J Am Coll Surg. 2020;231(1):150–4.10.1016/j.jamcollsurg.2019.12.044. 10.1016/j.jamcollsurg.2019.12.044 32081750

[8] Piraccini E, Biondi G, Corso RM, Maitan S. The use of rhomboid intercostal block, parasternal block and erector spinae plane block for breast surgery. J Clin Anesth. 2020;59:10.10.1016/j.jclinane.2019.06.004. 10.1016/j.jclinane.2019.06.004 31176954

[9] Kim DH, Oh YJ, Lee JG, Ha D, Chang YJ, Kwak HJ. Efficacy of Ultrasound-Guided Serratus Plane Block on Postoperative Quality of Recovery and Analgesia After Video-Assisted Thoracic Surgery: A Randomized, Triple-Blind, Placebo-Controlled Study. Anesth Analg. 2018;126(4):1353–61.10.1213/ane.0000000000002779. 10.1213/ANE.0000000000002779 29324496

[10] Toda C, Gupta RK, Elsharkawy H. Rhomboid Intercostal Block Combined With Interscalene Nerve Block for Sternoclavicular Joint Reconstruction. Ochsner J. 2021;21(2):214–6.10.31486/toj.20.0020. 10.31486/toj.20.0020 PMC8238101 34239386

[11] Elsharkawy H, Saifullah T, Kolli S, Drake R. Rhomboid intercostal block. Anaesthesia. 2016;71(7):856–7.10.1111/anae.13498. 10.1111/anae.13498 27291611

[12] Rahimzadeh P, Imani F, Faiz SHR, Boroujeni BV. Impact of the Ultrasound-Guided Serratus Anterior Plane Block on Post-Mastectomy Pain: A Randomised Clinical Study. Turk J Anaesthesiol Reanim. 2018;46(5):388–92.10.5152/tjar.2018.86719. 10.5152/TJAR.2018.86719 PMC6157977 30263863

[13] Lin J, Hoffman T, Badashova K, Motov S, Haines L. Serratus Anterior Plane Block in the Emergency Department: A Case Series. Clin Pract Cases Emerg Med. 2020;4(1):21–5.10.5811/cpcem.2019.11.44946. 10.5811/cpcem.2019.11.44946 PMC7012558 32064417

[14] Moher D, Hopewell S, Schulz KF, Montori V, Gøtzsche PC, Devereaux PJ, et al. CONSORT 2010 explanation and elaboration: updated guidelines for reporting parallel group randomised trials. International journal of surgery (London, England). 2012;10(1):28–55.10.1016/j.ijsu.2011.10.001. 10.1016/j.ijsu.2011.10.001 22036893

[15] DeLoach LJ, Higgins MS, Caplan AB, Stiff JL. The visual analog scale in the immediate postoperative period: intrasubject variability and correlation with a numeric scale. Anesth Analg. 1998;86(1):102–6.10.1097/00000539-199801000-00020. 10.1097/00000539-199801000-00020 9428860

[16] Otani K, Waterman B, Faulkner KM, Boslaugh S, Burroughs TE, Dunagan WC (2009). Patient satisfaction: focusing on "excellent". J Healthc Manag..

[17] Dahl JB, Møiniche S. Pre-emptive analgesia. Br Med Bull. 2004;71:13–27.10.1093/bmb/ldh030. 10.1093/bmb/ldh030 15596866

[18] Blanco R, Parras T, McDonnell JG, Prats-Galino A. Serratus plane block: a novel ultrasound-guided thoracic wall nerve block. Anaesthesia. 2013;68(11):1107–13.10.1111/anae.12344. 10.1111/anae.12344 23923989

[19] Zhang JG, Jiang CW, Deng W, Liu F, Wu XP. Comparison of Rhomboid Intercostal Block, Erector Spinae Plane Block, and Serratus Plane Block on Analgesia for Video-Assisted Thoracic Surgery: A Prospective, Randomized, Controlled Trial. Int J Clin Pract. 2022;2022:6924489.10.1155/2022/6924489. 10.1155/2022/6924489 PMC9246596 35832798

[20] Ökmen K, Köprücüoğlu M. Rhomboid Intercostal and Serratus Anterior Interfascial Plane Blocks for the Treatment of Post-Operative Pain after Video-Assisted Thoracoscopic Surgery: A Retrospective Propensity-Matched Study. Turk J Anaesthesiol Reanim. 2021;49(3):211–7.10.5152/tjar.2020.471. 10.5152/TJAR.2020.471 PMC10335716 35110140

[21] Elsharkawy H, Maniker R, Bolash R, Kalasbail P, Drake RL, Elkassabany N. Rhomboid Intercostal and Subserratus Plane Block: A Cadaveric and Clinical Evaluation. Reg Anesth Pain Med. 2018;43(7):745–51.10.1097/aap.0000000000000824. 10.1097/AAP.0000000000000824 30169476

[22] Hetta DF, Rezk KM. Pectoralis-serratus interfascial plane block vs thoracic paravertebral block for unilateral radical mastectomy with axillary evacuation. J Clin Anesth. 2016;34:91–7.10.1016/j.jclinane.2016.04.003. 10.1016/j.jclinane.2016.04.003 27687353

[23] Kunhabdulla NP, Agarwal A, Gaur A, Gautam SK, Gupta R, Agarwal A (2014). Serratus anterior plane block for multiple rib fractures. Pain Physician.

[24] Deng W, Hou XM, Zhou XY, Zhou QH. Rhomboid intercostal block combined with sub-serratus plane block versus rhomboid intercostal block for postoperative analgesia after video-assisted thoracoscopic surgery: a prospective randomized-controlled trial. BMC Pulm Med. 2021;21(1):68.10.1186/s12890-021-01432-7. 10.1186/s12890-021-01432-7 PMC7908696 33632189

[25] Altıparmak B, Korkmaz Toker M, Uysal AI, Dere Ö, Uğur B. Evaluation of ultrasound-guided rhomboid intercostal nerve block for postoperative analgesia in breast cancer surgery: a prospective, randomized controlled trial. Reg Anesth Pain Med. 2020;45(4):277–82.10.1136/rapm-2019-101114. 10.1136/rapm-2019-101114 32079739

[26] Park MH, Kim JA, Ahn HJ, Yang MK, Son HJ, Seong BG. A randomised trial of serratus anterior plane block for analgesia after thoracoscopic surgery. Anaesthesia. 2018;73(10):1260–4.10.1111/anae.14424. 10.1111/anae.14424 30120832

[27] Zhang X, Zhang C, Zhou X, Chen W, Li J, Wang H, et al. Analgesic Effectiveness of Perioperative Ultrasound-Guided Serratus Anterior Plane Block Combined with General Anesthesia in Patients Undergoing Video-Assisted Thoracoscopic Surgery: A Systematic Review and Meta-analysis. Pain Med. 2020;21(10):2412–22.10.1093/pm/pnaa125. 10.1093/pm/pnaa125 32488265

